# HIV Rapid Diagnostic Test Inventories in Zambézia Province, Mozambique: A Tale of 2 Test Kits

**DOI:** 10.15171/ijhpm.2019.07

**Published:** 2019-02-26

**Authors:** Christopher C. Wahlfeld, Amina Muicha, Paulo Harrison, Aaron M. Kipp, Gael Claquin, Wilson P. Silva, Ann F. Green, C. William Wester, Troy D. Moon

**Affiliations:** ^1^Vanderbilt Institute for Global Health, Vanderbilt University Medical Center, Nashville, TN, USA.; ^2^Friends in Global Health, Department of Pharmacy, Quelimane, Mozambique.; ^3^Provincial Health Directorate, Quelimane, Mozambique.; ^4^Vanderbilt Institute for Global Health, Department of Medicine, Division of Epidemiology, Vanderbilt University Medical Center, Nashville, TN, USA.; ^5^Friends in Global Health, Maputo, Mozambique.; ^6^Vanderbilt Institute for Global Health, Department of Medicine, Vanderbilt University Medical Center, Nashville, TN, USA.; ^7^Vanderbilt Institute for Global Health, Department of Pediatrics, Vanderbilt University Medical Center, Nashville, TN, USA.

**Keywords:** HIV, Counseling and Testing, Supply Chain System, Health System Strengthening, Mozambique

## Abstract

**Background:** The first pillar of the UNAIDS 90-90-90 goal seeks to accurately identify persons living with HIV (PLHIV), a process that is predicated on facilities having the necessary HIV tests available to perform the task. In many rural settings, the identification of PLHIV is accomplished through a two-step process involving the sequential use of 2 separate rapid diagnostic tests (RDTs). Inadequate inventory of either test has ramifications for the success of HIV-related programs. The purpose of this study was to evaluate the inventory levels of HIV RDT kits at specific healthcare facilities in Zambézia province, Mozambique.

**Methods:** Using facility-level pharmacy stock surveillance data from October 2015 through September 2016, we assessed the inventory levels of HIV RDTs at 75 health facilities in 8 districts within Zambézia province, Mozambique. Using programmatically established categories (good, sufficient, threatened, or stockout), defined in conjunction with the provincial health authorities, descriptive statistics were performed to determine inventory control of HIV RDTs at the district and health facility levels. Monthly proportions of adequate (good + sufficient) inventory were calculated for each district to identify inventory trends over the evaluation period. To assess whether the proportion of inadequate stocks differed between RDT, a mixed-effects logistic regression was conducted, with inadequate inventory status as the outcome of interest.

**Results:** When viewed as a whole, the inventory of each test kit was reported as being at adequate levels more than 89% of the time across the 75 facilities. However, disaggregated analysis revealed significant variability in the inventory levels of HIV RDTs at the district level. Specifically, the districts of Inhassunge, Namacurra, and Pebane reported inadequate inventory levels (threatened + stockout), of one or both test kits, for more than 10% of the study period. In addition, a disparity between inventory levels of each test kit was identified, with the odds of reporting inadequate inventory levels of the confirmatory test (Uni-Gold™) being approximately 1.8-fold greater than the initial test (Determine™) (odds ratio: 1.82, 95% CI: 1.40-2.38).

**Conclusion:** As Test and Treat programs evolve, a significant emphasis should be placed on the "test" component of the strategy, beginning with assurances that health facilities have the adequate inventory of RDT necessary to meet the needs of their community. As national policy-makers rely predominantly on data from the upstream arm of the supply chain, it is unlikely the disparity between inventory levels of HIV RDTs identified at individual districts and specific health facilities would have been recognized. Moving forward, our findings point to a need for (1) renewed efforts reinforcing appropriate downstream forecasting of essential medicines and diagnostic tests in general and for Uni-Gold™ test kits specifically, and (2) simple metrics that may be routinely collected at all health facilities and which may then easily and quickly flow upstream so that policy-makers may optimally allocate resources.

## Background

Diagnostic testing is a cornerstone of public health efforts to detect infection and acts as the fundamental precursor to prevention, treatment, and disease management. The significance of diagnostic testing has been underscored by the Sustainable Development Goal of ending the HIV/AIDS epidemic by 2030.^1,2^ Efforts to achieve this goal are embodied by programs such as the World Health Organization’s “Test-and-Treat” strategy, which advocates for all individuals who test positive for HIV being placed on life-long combination antiretroviral therapy (ART), regardless of their CD4+ cell count.^3,4^ This proactive approach has been endorsed by the UNAIDS’ 90-90-90 initiative.^5^ In an effort to reduce HIV to a low-level endemic disease, the 90-90-90 strategy seeks to have 90% of persons living with HIV (PLHIV) aware of their status by 2020; 90% of all individuals diagnosed with HIV placed on sustained ART; and 90% of individuals who have initiated ART achieving virologic suppression.^5,6^ The success, or failure, of these ambitious programs rests upon the vast majority of PLHIV knowing their status, a pursuit which is incumbent upon accurate diagnostic testing.

The number of PLHIV continues to grow, increasing from 36.7 million in 2015 to 36.9 million in 2017; however, of those infected, it has been estimated that 25%-33% remain unaware of their HIV status.^5-8^ To address this gap and achieve the goal of 90% of PLHIV knowing their status, HIV testing must be scaled-up to unprecedented levels.^6,9^ Rapid diagnostic tests (RDTs) for HIV play an instrumental role in this process, providing a low-cost approach to HIV screening without the need for laboratory-based equipment or highly trained technicians. Additionally, HIV RDTs have the benefit of providing results within 30 minutes, thereby allowing for testing, counseling, and referral to take place during a single visit.^10,11^

Ensuring a consistent and continuous flow of HIV RDTs, in appropriate quantities, at all points along the supply chain, is a complex process requiring a combination of accurate forecasting, procurement, and adequate in-country transportation and delivery services.^12-14^ Managing an adequate supply of HIV RDTs is further complicated by HIV screening algorithms. Unlike RDTs used to detect other medical conditions such as malaria, the algorithm for HIV testing requires the sequential use of 2 separate RDTs.^10^ When an individual is screened for HIV and the initial test results are negative, no further testing is necessary at that time. However, if the test results are positive, a second confirmatory test is required. This second test is performed using a different type of HIV RDT with a greater specificity.^15^ In cases where the initial test is positive and the confirmatory test is negative, protocols often dictate that the 2 RDT algorithm be repeated. Should the two-test algorithm remain indeterminant after repeat testing, patients are asked to come back 3-4 weeks later to repeat the testing algorithm.^15,16^

The absence of either RDT used in this testing sequence may result in missed opportunities to adequately capture the HIV status of at-risk persons and potentially delay the timely initiation of ART. While the stockout of either HIV RDT is a signal that the supply chain is failing, insufficient inventory levels should also be seen as an important indicator of a potentially fragile system.

### Setting

Located in north-central Mozambique, Zambézia province is an extremely poor region of Mozambique, estimated to be home to 5 million residents in 2017.^17-19^ A predominantly rural province with limited infrastructure, Zambézia was estimated to have an HIV prevalence of 15.1% among adults 15-49 years of age in 2015.^20^ As of 2016, Mozambique’s progress towards the 90-90-90 targets showed that 61% of its national HIV population had been diagnosed with HIV and of those 54% were receiving ART.^21,22^

### HIV Rapid Test Kit Supply Chain in Mozambique

HIV RDTs nationally approved for use in Mozambique consist of the Abbott Determine™ HIV 1/2 for initial testing and the Trinity Biotech Uni-Gold™ Recombigen^®^ HIV test for confirmatory testing. This initial and confirmatory testing algorithm is used as point-of-care testing at health facilities within their laboratories and when appropriate by providers at service delivery points. In addition, this algorithm is used within voluntary counseling and testing centers, at the community level for community HIV testing campaigns, and to test health facility blood supplies. Beyond HIV RDT, the only other method used in country for HIV diagnosis is by polymerase chain reaction in children less than nine-months of age, as part of the Early Infant Diagnosis strategies.

**Figure 1:**
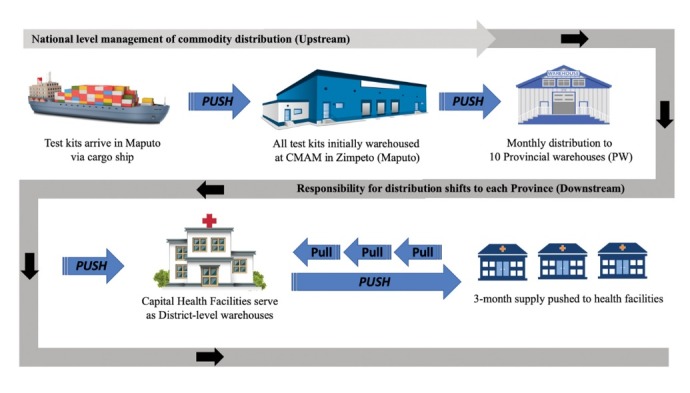
Distribution of HIV RDT Kits in Mozambique. Abbreviations: CMAM, Central Drugs and Medical Supplies Procurement; RDT, rapid diagnostic test.

The supply chain system for HIV RDTs in Mozambique begins with the Central Drugs and Medical Supplies Procurement (CMAM) service, which is responsible for national demand planning, procurement, and initial warehousing of all essential health products for the national health system.^23^ All purchased HIV RDTs arrive in Maputo (the nation’s capital) via cargo ship and are warehoused at the CMAM central warehouse in Zimpeto. From the CMAM warehouse, HIV RDTs are then distributed to each of the ten provincial warehouses, either by air freight or by truck, on a monthly basis (Figure 1).^24^ Once commodities arrive at the provincial warehouses, Mozambique’s supply chain becomes decentralized with the responsibility for management and distribution of commodities shifting further downstream to each Provincial Health Directorate (*Direcção Provincial de Saúde*, DPS) and their respective provincial pharmacy warehouses. From these provincial warehouses, commodities are pushed out to their respective district warehouses which then allocate supply to individual health facilities through a push-pull strategy.^25^ Unlike the distribution of commodities upstream, the process of distributing commodities downstream is typically accomplished through a series of diverse, often irregular, transport options.

Protocol dictates that health facilities have a one-month buffer of inventory for HIV RDTs at all times. To calculate the quantity of inventory to be requested, monthly consumption is multiplied by 2, and inventory on hand is subtracted from this amount.^26^ Assuming that sufficient inventory is available upstream, the quantity of test kits distributed to each health facility is based upon monthly consumption reports and inventory data that health facilities provide to their district warehouse. The quantity of test kits allocated to a district-level warehouse is, in turn, based upon the reports the district provides to the provincial warehouse. This process continues upstream, ultimately guiding the quantity of test kits ordered by CMAM.^26^

### Technical Support and Monitoring of District Health Facility Pharmacies

Friends in Global Health, a Mozambican-registered non-governmental organization, affiliated with the Vanderbilt Institute for Global Health has been working in Zambézia province since 2007. As part of its current activities, a multidisciplinary team of technical advisors from Friends in Global Health provides support and mentoring to a large number of health facilities across multiple districts within Zambézia province (n = 115 in 12 districts during the study period). These 12 districts have a high disparity of reported HIV prevalence, with variation in positive testing rates reported in routine programmatic data depending on the context in which HIV testing occurred.

Visiting each health facility once a month, on average, technical assistance related to managing stocks of HIV RDTs includes warehouse and/or pharmacy supply room organization, planning and coordination of supply chain logistics with health facilities, documentation of quality measures related to stock utilization, and the timely and appropriate completion of requisitions. This level of individual health facility focused technical support is not part of routine activities for all health facilities nationwide.

While visiting health facilities, mentoring teams conduct exercises to verify records, documenting the available stock at each health facility through the counting of physical inventory. On weeks when the multidisciplinary mentorship team was not present at a particular site, health facility pharmacists are contacted by phone to verify that the information in the mentoring team’s tracking spreadsheet reflect the stock levels reported by the on-site pharmacist for that week.

In 2008, the international community launched a rallying cry to “*Know your epidemic, Know your response,*” calling on program implementers to move away from standard approaches and to adapt their programs to individual community or health facility needs.^27^ To date, few studies have evaluated HIV RDT stock trends in Mozambique, particularly within the more downstream segments of the supply chain. The objective of this study was to “Know our epidemic” by describing trends over time in HIV RDT inventories at select health facilities in Zambézia province, Mozambique, with the aim of identifying patterns of threatened inventory levels and/or stockouts of the RDTs. By identifying these patterns further downstream in the supply chain, we sought to aid national policy-makers’ efforts to optimize resources as they strive to meet Mozambique’s 90-90-90 targets by 2020.

## Methods

### Data Collection

Mirroring the timeline employed by provincial health authorities to report on HIV data to the Ministry of Health and to programs such as PEPFAR, a serial cross-sectional study was conducted with the intent of assessing the availability of Determine™ and Uni-Gold™ test kits from October 2015 through September 2016 using pharmacy stock surveillance records from 75 supported health facilities in 8 districts within Zambézia province.

**Figure 2:**
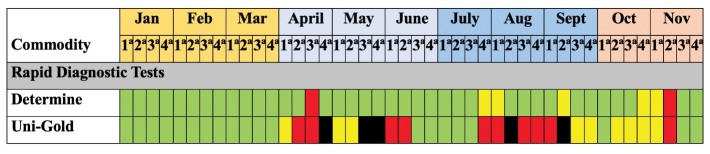
Illustrative Heat Map of HIV RDT Kit Commodities. Data comes from one health facility illustrating the tracking sheets used at all data collection sites involved in this study. Abbreviation: RDT, rapid diagnostic test.

Pharmacy stock surveillance records track the categorical status of essential medications and test kits by placing each commodity into one of 4 programmatically defined categories: good, sufficient, threatened, or stockout. The category of “good,” indicated that inventory levels of a specific commodity not only met the forecasted need for the week but also maintained the recommended 1-month buffer for that product. A “sufficient” status indicated that a health facility had the necessary inventory on hand to meet the forecasted need for that week, however the 1-month additional supply was not maintained. A commodity was categorized as “threatened” if a supply of the commodity existed but the inventory on hand had fallen below the forecasted need for the week. Finally, the category “stockout” indicated that the product was completely unavailable. These categories were visually expressed within the stock surveillance tracking sheets as a 4 color “heat map” with “good” shown in green, “sufficient” shown in yellow, “threatened” in red, and “stockout” in black (Figure 2).

As part of the data cleaning process, the decision was made to exclude health facilities from the study if reportable data were missing for more than 6 consecutive weeks. After taking this step, the tracking spreadsheets from the remaining health facilities were consistently missing data for one or more weeks in December. Therefore, the entire month of December was removed from the sample. After exclusion, the final study sample consisted of 11 months of data for 75 supported facilities across 8 districts. For this remaining sample, unreported data was a rarity, accounting for less than 1% of the study period. Prior to analysis, data from the original Excel spreadsheets were selected at random and compared to the data imported into STATA to ensure no errors were introduced.

### Data Analysis

Descriptive statistics were performed to determine inventory status (good, sufficient, threatened, stockout) for the overall sample, as well as at the district and facility levels. For the purposes of this evaluation we defined “adequate stock” by combining the categories of good and sufficient stock levels. Similarly, the combination of threatened and stockout inventory levels were used to define the category “inadequate stock.” Inventory levels of Determine™ and Uni-Gold™ test kits were assessed separately. As no prior measure for assessing inventory levels of categorical data existed, we chose an inadequate stock threshold of 10% to identify districts and individual health facilities of concern.

To assess patterns of inventory status at the district level over time, weekly facility-level data were collapsed into dichotomous categories for each month. The number of health facilities within each district identified as being inadequate were counted and the monthly counts were divided by the total number of facilities within the district to create the proportion of facilities within the district that experienced inadequate inventory levels each month. To assess whether the proportion of inadequate stocks differed between Determine™ and Uni-Gold™ kits, while accounting for the nested structure of the data, a mixed-effects logistic regression was conducted, with inadequate inventory status as the outcome of interest. Covariates included type of test kit and time (month) with random effects for district and health facility. STATA 14 was used to conduct all statistical analysis.

## Results

### Overall Sample

During the 44-week period that inventory levels of Determine™ and Uni-Gold™ test kits were tracked, a total of 3277 weekly inventory observations were reviewed.

When examining the study sample as a whole, inventory levels of Determine™ test kits were reported as being at adequate levels (good + sufficient) more than 90% of the time. Threatened inventories of Determine™ RDTs were reported 5% of the time, with instances of stockouts reported only 4 times (0.1%) across all 8 districts over the study period, suggesting a high level of commodity security for Determine™ RDTs across the sample (Table 1). However, an examination of Uni-Gold™ RDT inventory revealed a much higher frequency of inadequate stock, with levels reaching 10.3% (threatened stock (7.6%) and stockout (2.7%), respectively), for the confirmatory RDT.

**Table 1:** Categorical Distribution of HIV RDT Inventory Levels Reported in Zambézia Province

**(# Health Facilities in District)** **(# Weekly Inventory Records)**	**Good** **No. (%)**	**Sufficient** **No. (%)**	**Threatened** **No. (%)**	**Stockout** **No. (%)**	Inadequate* **No. (%)**
Combined sample (n = 75)					
Determine™ (3277)	2491 (76.0)	618 (18.9)	164 (5.0)	4 (0.1)	168 (5.1)
Uni-Gold™ (3277)	2139 (65.3)	801 (24.4)	250 (7.6)	87 (2.7)	340 (10.3)
Alto Molocue (n = 12)					
Determine™ (528)	347 (65.7)	141 (26.7)	40 (7.6)	0 (0)	40 (7.6)
Uni-Gold™ (528)	319 (60.4)	189 (35.8)	16 (3.0)	4 (0.8)	20 (3.8)
Gilé (n = 8)					
Determine™ (336)	301 (89.6)	23 (6.9)	12 (3.6)	0 (0)	12 (3.6)
Uni-Gold™ (336)	275 (81.9)	46 (13.7)	15 (4.5)	0 (0)	15 (4.5)
Inhassunge (n = 5)					
Determine™ (220)	17 (7.7)	186 (84.6)	16 (7.3)	1 (0.5)	17 (7.7)
Uni-Gold™ (220)	15 (6.8)	105 (47.7)	67 (30.5)	33 (15)	100 (45.5)
Maganja da Costa (n = 5)					
Determine™ (218)	172 (78.9)	43 (19.7)	3 (1.4)	0 (0)	3 (1.4)
Uni-Gold™ (218)	52 (23.9)	152 (69.7)	7 (3.2)	7 (3.2)	14 (6.4)
Mopeia (n = 9)					
Determine™ (396)	365 (92.2)	23 (5.8)	8 (2.0)	0 (0)	8 (2.0)
Uni-Gold™ (396)	334 (84.3)	40 (10.1)	21 (5.3)	1 (0.3)	22 (5.5)
Morrumbala (n = 18)					
Determine™ (788)	572 (72.6)	154 (19.5)	61 (7.7)	1 (0.1)	62 (7.9)
Uni-Gold™ (788)	649 (82.4)	105 (13.3)	30 (3.8)	4 (0.5)	25 (3.2)
Namacurra (n = 7)					
Determine™ (307)	298 (97.1)	3 (1.0)	4 (1.3)	2 (0.7)	6 (2.0)
Uni-Gold™ (307)	183 (59.6)	87 (28.3)	26 (8.5)	11 (3.6)	37 (12.1)
Pebane (n = 11)					
Determine™ (484)	419 (86.6)	45 (9.3)	20 (4.1)	0 (0)	20 (4.1)
Uni-Gold™ (484)	312 (64.5)	77 (15.9)	68 (14.1)	27 (5.6)	95 (19.6)

Abbreviation: RDT, rapid diagnostic test. * Inadequate stock = Threatened Stock + Stockout.

### District Level

The 4 reported stockouts of the Determine™ RDT occurred in 3 supported districts; namely, Morrumbala (n = 1), Inhassunge (n= 1), and Namacurra (n = 2). However, all 8 districts reported experiencing threatened inventory levels of the Determine™ test kit at some point during the study period, with threats occurring 1.3% to 7.7% of the time. Despite these threats, no individual district exceeded the 10% threshold for inadequate inventories of Determine™.

In contrast, stockouts of the Uni-Gold™ RDT were widespread, with 7 of the supported 8 districts reporting stockouts during the study period. The district of Gilé was a notable exception, with no reported stockouts of either test kit. Similar to inventory levels of the Determine™ RDT, all 8 districts reported threatened levels of Uni-Gold™ at some point during the study period. In turn, the frequency of districts reporting threatened inventory levels were comparatively higher for Uni-Gold™, occurring between 3.0% and 30.5% of the time. Three supported districts were identified as exceeding the 10% threshold of concern for inadequate inventory levels of Uni-Gold™: Namacurra (12.1%), Pebane (19.6%) and Inhassunge (45.5%).

The district of Inhassunge stood out as being of particular concern, reporting good inventory levels of Uni-Gold™ merely 6.8% of the time and sufficient levels of stock only 47.7% of the time. In other words, inventory levels of Uni-Gold™ were reported as being adequately stocked for just over half of the study period (54.5%). Maintenance of the prescribed 1-month buffer of RDTs in Inhassunge was also noted as a challenge for Determine™ RDTs with good levels of the RDT reported less than 8% of the time. However, sufficient inventory levels were reported 84.6% of the time.

### Health Facility Level

The frequency of inventory status was examined further downstream, at the health facility level, to assess variability across facilities within each of the 8 supported districts. Although district level assessments did not reveal inadequate inventory levels of Determine™ breaching our predefined 10% threshold, facility level assessments revealed 11 health facilities reporting inadequate inventory levels of Determine™ that exceeded the threshold. These health facilities were located within the districts of Alto Molocue (n = 4), Inhassunge (n = 2), and Morrumbala (n = 5), (Supplementary file 1).

Assessment of Uni-Gold™ inventories revealed a more widespread occurrence of inadequate inventory levels in excess of the 10% threshold, identifying a total of 25 health facilities across 6 supported districts (Table 2). All 11 health facilities within the district of Pebane exceeded the threshold, reporting inadequate levels of Uni-Gold™ between 11.4% to 27.3% of the time. The highest frequency was reported at the capital health facility, which houses the district-level warehouse. It is important to note, however, that the primary driver pushing health facilities in Pebane beyond the 10% threshold was the high percentage of threatened inventories reported, as opposed to reports of stockouts.

**Table 2:** Inadequate Inventory Levels of the Uni-Gold™ RDT Exceeding the 10% Threshold

**District (# Health Facilities)**	**Test Kit**	**Threatened**	**Stockout**	Inadequate*
Inhassunge (n = 5)				
CHF	Uni-Gold™	27.3%	9.1%	36.4%
Bingagira	Uni-Gold™	38.6%	13.6%	52.3%
Chirimane	Uni-Gold™	20.5%	18.2%	38.6%
Gonhane	Uni-Gold™	38.6%	18.2%	56.8%
Palane Mucula	Uni-Gold™	27.3%	15.9%	43.2%
Maganja da Costa (n = 5)				
Mbala	Uni-Gold™	2.3%	9.1%	11.4%
Mopeia (n = 9)				
CHF	Uni-Gold™	13.6%	0.0%	13.6%
Lua Lua	Uni-Gold™	18.2%	0.0%	18.2%
Morrumbala (n = 18)				
DDM Morrumbala	Uni-Gold™	30.2%	9.3%	39.5%
Reis	Uni-Gold™	11.4%	0.0%	11.4%
Namacurra (n = 7)				
CHF	Uni-Gold™	18.2%	9.1%	27.3%
Macuse	Uni-Gold™	11.4%	2.3%	13.6%
Mbawa	Uni-Gold™	9.1%	2.3%	11.4%
Muebele	Uni-Gold™	6.8%	6.8%	13.6%
Pebane (n = 11)				
CHF	Uni-Gold™	20.5%	6.8%	27.3%
7 de Abril	Uni-Gold™	11.4%	9.1%	20.5%
Alto Maganha	Uni-Gold™	15.9%	6.8%	22.7%
Impaca	Uni-Gold™	11.4%	0.0%	11.4%
Magiga	Uni-Gold™	15.9%	6.8%	22.7%
Malema	Uni-Gold™	13.6%	6.8%	20.4%
Mulela	Uni-Gold™	13.6%	6.8%	20.4%
Muligode	Uni-Gold™	15.9%	0.0%	15.9%
Naburi	Uni-Gold™	13.6%	4.5%	18.1%
Pele Pele	Uni-Gold™	13.6%	6.8%	20.4%
Tomeia	Uni-Gold™	9.1%	6.8%	15.9%

Abbreviations: CHF, Capital Health Facility; RDT, rapid diagnostic test. * Inadequate stock = Threatened Stock + Stockout.

A similar pattern was noted within the district of Inhassunge. All 5 health facilities in the district exceeded the 10% threshold of concern, reporting inadequate levels of Uni-Gold™ ranging between 36.4% and 56.8%. Additionally, it was noted that 4 of the 5 facilities (80%) within the district of Inhassunge exceeded the 10% threshold when looking at complete stockouts alone.

In contrast to the global patterns of inadequate stock levels reported at all health facilities in the other 2 districts in which the 10% threshold was breached, namely Pebane and Inhassunge, only 4 of 7 (57%) health facilities in the district of Namacurra reported inadequate inventory levels, with Uni-Gold™ exceeding the 10% threshold, ranging between 11.4% and 27.3%. The highest of these levels was reported by the district capital health facility.

### Patterns Over Time

**Figure 3:**
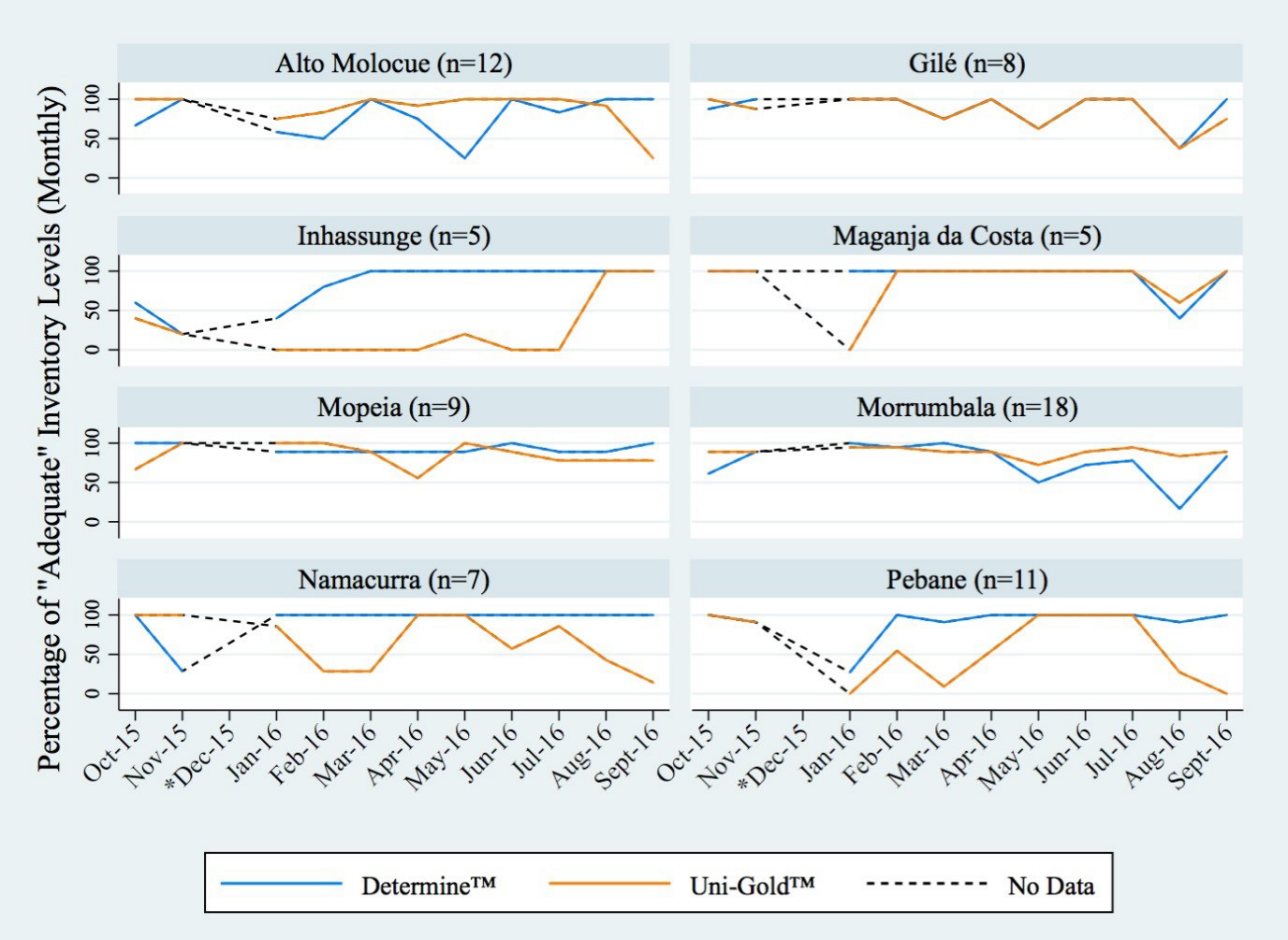
Percentage of Adequate Inventory Levels for Each District Over Time. * The month of December was removed from the study sample to address consistently missing data from all facilities.

We further sought to describe adequate test kit inventory levels over the 11-month study period. Graphs generated to depict the percentage of adequate inventory levels by month revealed various degrees of inventory fluctuation within each district (Figure 3). Inconsistent inventory levels of the Determine™ RDT were noted in the districts of Alto Molocue and to a lesser extent Morrumbala. However, when compared with inventory levels of the Uni-Gold™ test kit, Determine™ test kit inventory levels, in the remaining districts, were shown to be maintained at consistently high levels throughout the study period. Comparatively, adequate inventory levels of the Uni-Gold™ test kit were shown to be particularly erratic in the districts of Namacurra and Pebane, whereas the district of Inhassunge was seen as experiencing persistently inadequate levels of Uni-Gold™ throughout the majority of the study period.

As these test kits are used sequentially, it is important to note the disparity between levels of adequate inventory of the 2 test kits within a district during a given month. Results from the mixed-effect model confirmed the disparity between test kit inventories noted above, revealing an 82% higher odds of reporting inadequate inventory levels for the Uni-Gold™ test kit than for Determine™ (adjusted odds ratio of 1.82, 95% CI: 1.40-2.38).

## Discussion

Our evaluation of HIV RDT inventory levels at health facilities within Zambézia province, indicate that Determine™ test kits were generally well maintained across the majority (ie, >90%) of the study sample. Inventory levels of the confirmatory Uni-Gold™ test kits, however, told a different tale. When we began to closely evaluate stock levels disaggregated by district, and then by health facility within districts, we identified stark disparities between the inventory levels of the 2 test kits and a much higher than anticipated frequency of threatened stocks for Uni-Gold™.

We ultimately identified 3 districts of concern: namely, the districts of Pebane, Inhassunge, and Namacurra. Inhassunge stood out as a particularly vulnerable district. This was especially worrisome as Inhassunge is the district closest to Quelimane city, the capital of Zambézia province and the location of the provincial warehouse. As such, Inhassunge should have sufficient access to, and communication with, the provincial warehouse to be able to avoid the high levels of stockouts that were reported.

Though missing data is a concern across the sample, the district of Gilé stood out as a potential success story. Gilé did not report stockouts of either test kit and reported threatened inventory levels of less than 5% during the period of evaluation. When looking at the proportion of adequate inventory levels reported across the facilities of Gilé over time, it is noted that the inventory levels of Determine™ and Uni-Gold™ mirror each other throughout the study period, suggesting a robust and stable forecasting system. As a mid-sized district with 8 supported health facilities, Gilé warrants further investigation into best practices that may be applied to other districts.

Our study is not the first to document such a disparity in RDT inventory levels. An assessment of HIV test kit inventories in Tanzania during the early 2000s revealed that in health facilities offering HIV testing, often only one type of test kit was available.^12^ More recently, research conducted in Ethiopia in 2013 identified increased stockouts of Uni-Gold™ as compared to other HIV diagnostic test kits. However, as their analysis was more heavily focused on the stocks of antiretroviral medications, the authors did not comment as to the potential causes of this HIV test kit disparity.^28^ Lastly, an evaluation conducted by the Global Fund to fight AIDS, Tuberculosis and Malaria in the Democratic Republic of Congo from January 2015 to March 2016, found that the average number of days, over the 15-month study period, for stockouts of Uni-Gold™ was 59 days compared to just 7 days for Determine.^29^

The specific characteristics of different HIV RDTs, and the way they are utilized throughout HIV counseling and testing programs, pose particular challenges to forecasting and accurately quantifying the number of each test that will be required at an individual health facility.^11^ Though primarily limited in focus to the central, regional, and provincial (upstream) levels of Mozambique’s supply chain system, USAID´s Deliver Project has been working with CMAM, providing technical assistance to overcome the many barriers that can impact stock quantification exercises. From 2007 to 2015, significant work went into developing a logistics management information system, to improve the quality and use of consumption data for forecasting, and in training sufficient numbers of personnel on how to proficiently use the system.^30^

As previously described, Mozambique’s supply chain system is currently fragmented into 2 distinct arms. The portion starting at the provincial warehouses, which supplies various districts, and ultimately each individual health facility, represents the downstream arm, which is administratively managed through each Provincial Health Directorate. As stock levels of the Determine™ test kit were adequately maintained throughout the study period and districts, such as Gilé, were able to maintain adequate, mirrored levels of both HIV RDTs over time, we conclude that the HIV RDT stock disparities seen during the study period were not likely due to problems in distribution from the provincial warehouse to the district warehouses, nor from the district warehouses to the individual health facilities. Rather, the low levels of Uni-Gold™ stock experienced across our study facilities likely represents problems in individual health facility forecasting of their Uni-Gold™ needs.

This study has several limitations. This is not a multi-year study, with analysis limited to an 11-month time period. The use of categorical, composite stock data, as opposed to individual, raw numbers, inhibited our ability to assess inventory levels in relation to stock on hand, test kit consumption, and any inventory losses or adjustments that may impact forecasting. As of 4 of the 12 supported districts were excluded from analysis, due to missing data exceeding the study’s inclusion criteria, our findings may not reflect the overall HIV RDT inventory status within Zambézia province. Additionally, as we did not have inventory data at the provincial level, we were unable to rule out the possibility that disparities between inventory levels of Determine™ and Uni-Gold™ test kits were not an artifact of supply chain issues occurring further upstream.

## Conclusion

In Mozambique, and beyond, it is important not to forget the importance of diagnostic testing. As Test-and-Treat strategies, such as the UNAIDS 90-90-90 initiative, continue to scale-up, the demand for HIV test kits will only increase, furthering the need for adequate forecasting and distribution. Our evaluation of 75 health facilities across 8 districts in Zambézia province, Mozambique, provided a snapshot of HIV RDT inventory status in a geographically rural setting.

By relying predominantly on composite data, produced from the upstream arm of the supply chain, current data reporting procedures make it easy for national policy-makers to interpret downstream inventory levels as being satisfactorily maintained. However, by focusing downstream, our study identified individual districts and specific health facilities exhibiting patterns of test kit inventory levels that require further attention, particularly if the goal to halt the HIV epidemic is to be achieved. Moving forward, our findings point to a need for (1) renewed efforts to reinforce health facility forecasting of essential medicines and diagnostic tests in general and for Uni-Gold™ test kits specifically, and (2) simple metrics that are routinely collected in all health facilities and that flow upstream easily and quickly so that policy-makers can optimize resource allocations.

## Ethical issues

This study was approved by the Vanderbilt University Institutional Review Board (160549); the Institutional Research Ethics Committee for Health of Zambézia Province (Comité Institucional de Bioética para Saúde-Zambézia) (02-CIBS-Z-16); the Provincial Health Directorate of Health of Zambézia Province (Direcção Provincial de Saúde Zambézia) and the Centers for Disease Control and Prevention of Mozambique (2016-163).

## Competing interests

Authors declare that they have no competing interests.

## Authors’ contributions

CCW, CWW, AFG, and TDM were involved in study design. AM, PH, GC, and WPS were involved in data collection. CCW and AMK were responsible for cleaning and running statistical analysis. CCW, CWW, GC, AMK, WPS, and TDM were responsible for interpretation of results. CCW was responsible for writing the first draft. All authors contributed equally to editing draft versions and accept full responsibility for the content of the manuscript.

## Authors’ affiliations

^1^Vanderbilt Institute for Global Health, Vanderbilt University Medical Center, Nashville, TN, USA. ^2^Friends in Global Health, Department of Pharmacy, Quelimane, Mozambique. ^3^Provincial Health Directorate, Quelimane, Mozambique. ^4^Vanderbilt Institute for Global Health, Department of Medicine, Division of Epidemiology, Vanderbilt University Medical Center, Nashville, TN, USA. ^5^Friends in Global Health, Maputo, Mozambique. ^6^Vanderbilt Institute for Global Health, Department of Medicine, Vanderbilt University Medical Center, Nashville, TN, USA. ^7^Vanderbilt Institute for Global Health, Department of Pediatrics, Vanderbilt University Medical Center, Nashville, TN, USA.

## Supplementary files


Supplementary file 1 contains Table S1.

## Key messages

**Implications for policy makers**

- An assessment of HIV rapid diagnostic test (RDT) inventories in Zambézia province, Mozambique was conducted revealing a disparity in inventory levels between the initial and confirmatory tests at the district and facility levels, a finding that would have been lost if based upon routine reporting standards which emphasize only composite reporting of provincial level stocks.
- As demand for HIV testing increases, continued disparities in HIV RDT stocks threaten to undermine Mozambique’s feasibility to meet the first 90 of the UNAIDS 90-90-90 initiative, that of having 90% of HIV positive persons knowing their HIV status.

**Implications for public**

Our assessment of rapid diagnostic tests (RDTs) in Zambézia province, Mozambique revealed a disparity in the inventory levels of initial and confirmatory HIV RDTs, a finding that has implications for patient outcomes and on the success of initiatives seeking to end the HIV/AIDS epidemic. Inconsistencies in HIV RDT stocks at health facilities could lead to growing frustration among the communities served if individuals are unable to receive HIV testing on the day they arrive at the facility for an HIV test, potentially compounding poor trust in the facility as a resource and lowering health service access and demand.
