# Supplementary material for: HIV Rapid Diagnostic Test Inventories in Zambézia Province, Mozambique: A Tale of 2 Test Kits
Source: Int J Health Policy Manag. 2019 Feb 26;8(5):292–9. doi: 10.15171/ijhpm.2019.07 (PMC6571497; doi:10.15171/ijhpm.2019.07)
Supplement: Supplementary file 1 — contains Table S1. [file ijhpm-8-292-s001.pdf]

Supplemental File 1

Table S1. Inadequate Inventory Levels of the Determine™ RDT Exceeding the 10% Threshold

District (Total Facilities)	Test Kit	Threatened	Stockout	Inadequate Stock*
Alto Molocue (n=12)				
Gruveta	Determine™	13.6%	0.0%	13.6%
Muhia	Determine™	13.6%	0.0%	13.6%
Mutala	Determine™	11.4%	0.0%	11.4%
Novanana	Determine™	18.2%	0.0%	18.2%
Inhassunge (n=5)				
Bingagira	Determine™	11.4%	0.0%	11.4%
Gonhane	Determine™	11.4%	0.0%	11.4%
Morrumbala (n=18)				
CHF	Determine™	11.4%	0.0%	11.4%
Boroma	Determine™	9.1%	2.2%	11.3%
Chire	Determine™	18.2%	0.0%	18.2%
Megza	Determine™	11.4%	0.0%	11.4%
DDM Morrumbala	Determine™	23.2%	0.0%	23.2%

Abbreviations: CHF, Capital Health Facility; RDT, rapid diagnostic test.

*Inadequate stock = Threatened Stock + Stockout
